# Controversial Role of Kisspeptins/KiSS-1R Signaling System in Tumor Development

**DOI:** 10.3389/fendo.2018.00192

**Published:** 2018-04-30

**Authors:** Federica Fratangelo, Maria Vincenza Carriero, Maria Letizia Motti

**Affiliations:** ^1^IRCCS Istituto Nazionale Tumori “Fondazione G. Pascale”, Naples, Italy; ^2^Parthenope University of Naples, Naples, Italy

**Keywords:** kisspeptin, KiSS-1R, tumor development, tumor invasion, metastasis

## Abstract

*KiSS-1* was first described as a metastasis suppressor gene in malignant melanoma. *KiSS-1* encodes a 145 amino-acid residue peptide that is further processed, producing the 54 amino acid metastin and shorter peptides collectively named kisspeptins (KPs). KPs bind and activate KiSS-1R (GPR54). Although the KPs system has been extensively studied for its role in endocrinology of reproductive axis in mammals, its role in cancer is still controversial. Experimental evidences show that KP system exerts an anti-metastatic effect by the regulation of cellular migration and invasion in several cancer types. However, the role of KPs/KiSS-1R is very complex. Genomic studies suggest that KiSS-1/KiSS-1R expression might be different in the various stages of tumor development. Furthermore, overexpression of KiSS-1R has been reported to elicit drug resistance in triple negative breast cancer. In this review, we focused on multiple functions exerted by the KPs/KiSS-1R system in regulating tumor progression.

## Introduction

*KiSS-1* gene, located on human chromosome 1q32, encodes a precursor peptide of 145 amino acids that subsequently is processed by proteolytic cleavage into shorter peptides collectively defined as kisspeptins (KPs): KP-10, KP-13, KP-14, and KP-54 (metastin). KPs bind to G-protein-coupled receptor 54 (GPR54) also named KiSS-1R that activates the G proteins Gα_q/11_ (1, 2).

High levels of KiSS-1 have been found in the placenta and in the brain (3) and high expression levels of KiSS-1R were observed to in the placenta, pituitary, pancreas, and spinal cord (2). Low levels of KiSS-1R are present in lymph nodes, peripheral blood lymphocytes, adipose tissue, and spleen (1, 3).

**Figure 1:**
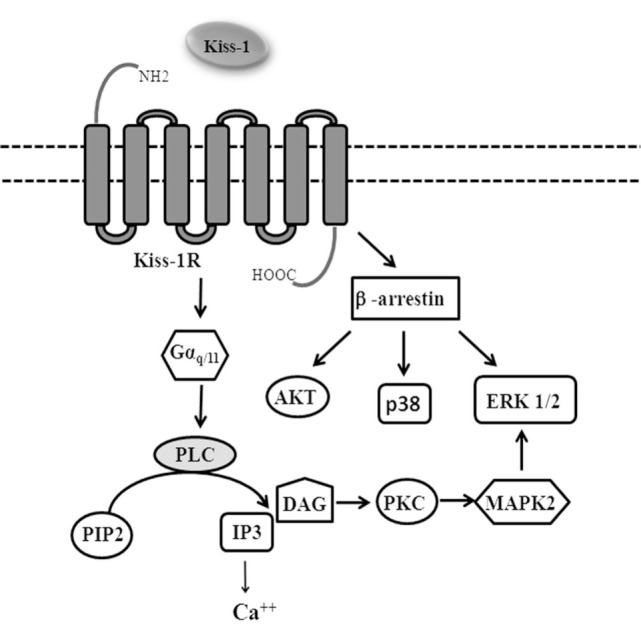
Signaling pathways activated by KiSS-1R.

KiSS-1/KiSS-1R coupled to Gα_q/11_ activates phospholipase C (PLC). PLC activation promotes the hydrolysis of phosphatidylinositol-4,5-bisphosphate which, in turn, leads to the production of two potential “second messengers” inositol-1,4,5-trisphosphate (IP3) and diacylglycerol (DAG). The activation of DAG leads to the activation of protein kinase C, ERK1/2, and p38 phosphorylation, while IP3 induces release of intracellular Ca^++^ (2, 4–6) (Figure 1).

G-protein-coupled receptors are regulated by β-arrestins, which not only desensitizes G-protein signaling, but also acts as molecular scaffolds and activates a series of signaling pathways including ERK 1/2, p38, PI3K/Akt, and cJun N-terminal kinase 3 (7, 8).

G-protein-coupled receptor serin/threonine kinase GRK2 and β-arrestins promote KiSS-1R desensitization by internalization *via* clathrin-coated pits (9, 10).

The anti-metastatic role of KP has been identified by early studies performed in melanoma and breast cancer cells before its role in regulating the reproductive functions. Although the anti-metastatic role has not been studies extensively as compared to the reproductive function, nevertheless a large body of literature documented the involvement of the KiSS-1/KiSS-1R system in supporting tumor progression. Furthermore, recent studies have demonstrated that plasma levels of KPs are increased in colorectal cancer (CRC) and small renal tumor patients, suggesting that KPs may be considered plasmatic biomarkers in these tumors (11, 12).

**Figure 2:**
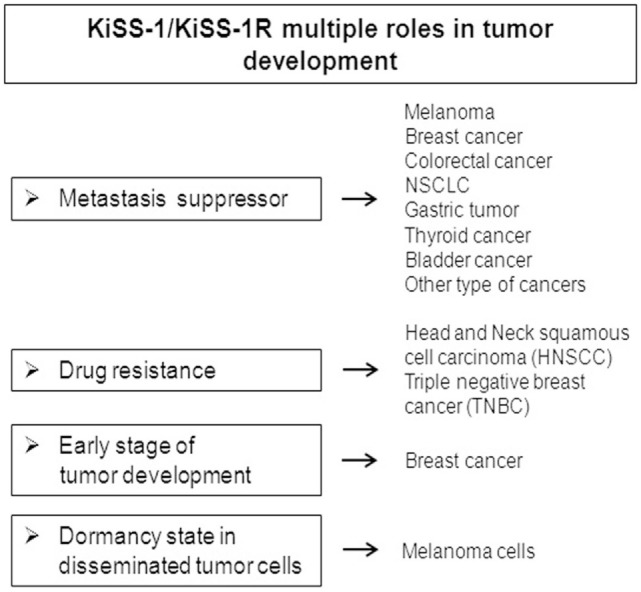
Multiple functions exerted by the KiSS-1/KiSS-1R system in regulating tumor progression.

In this review, we focused on multiple functions exerted by the KPs/KiSS-1R system in cancer (Figure 2).

## KiSS-1 as a Metastasis Suppressor Gene

### First Evidence in Melanoma and Breast Cancer

*KiSS-1* was primarily identified as a human melanoma metastasis suppressor gene using subtractive hybridization between the metastatic human melanoma cell line C8161 and non-metastatic variants generated after microcell-mediated transfer of chromosome 6 into C8161 cells to suppress their ability to metastasize (13, 14).

Transfection of *KiSS-1* into metastatic human melanoma cell lines suppressed metastasis in athymic nude mice by 50–95% (13). Thus, since *KiSS-1* map to the long arm of chromosome 1 and its expression suppresses metastasis of melanoma, it was hypothesized that some regulators of *KiSS-1* are encoded by chromosome 6 (14). Indeed, a region of 40cM between 6q16.3 and q23 was identified as an important regulatory region of *KiSS-1* (15). Moreover, Shirasaki and co-workers documented the loss of 6q16.3-q23 in more than 50% of melanoma metastases and that loss of heterozygosis of this region associates with the loss of *KiSS-1* (16).

Only 1 year after the first paper on melanoma, Lee and colleagues conducted another study on breast cancer demonstrating that human breast carcinoma MDA-MB-435 cells transfected with *KiSS-1* expression vector exhibit a lower metastatic, but not tumorigenic potential in comparison with control cells. This study was based on the observations that 1q chromosome is frequently deleted in late stage of human breast carcinomas and that *KiSS-1* is located on chromosome 1q32-q41 (17).

The studies that followed to better underline the anti-metastatic role of *KiSS-1* in breast cancer, instead, revealed the existence of controversial data for this type of tumors.

In fact a study conducted in 2005 by Martin and colleagues demonstrated that *KiSS-1* may not be functioning as a metastasis suppressor in breast cancer cells (18).

They found that the expression of *KiSS-1* mRNA had significantly increased in primary tumors in comparison with normal mammary tissues; they also found that levels of KiSS-1 expression were higher in metastatic disease patients compared to healthy individuals, and that this was associated with poor patient prognosis.

This study contrasted the reports which supported the anti-metastatic potential of *KiSS-1* in breast cancer. The analysis of *KiSS-1* mRNA levels in fresh frozen tissue samples from ductal invasive breast carcinomas revealed a significant reduction of KiSS-1 expression in brain metastases as compared to the primary tumors (19). These results were confirmed in 2012 by Ulasov and co-workers, who found a statistically significant downregulation of *KiSS-1* mRNA and protein in brain metastasis compared to primary tumors (20).

So, even though several studies have identified the *KiSS-1* gene as a metastasis suppressor in breast cancer, the existence of contradictory data underlines the need to better elucidate its biological role in this particular cancer.

Breast tumors are divided into estrogen receptor α (ERα)-positive and ERα-negative tumors and the role of KiSS-1 and KiSS-1R in the positive group are conflicting.

Estradiol (E2) through its receptor ERα is involved in mammary ductal normal growth and development. It is well established that estrogen is associated with increased breast cancer risk (21).

It has been demonstrated that E2 negatively regulates KiSS-1 and KiSS-1R expression. They showed that in ERα-positive primary tumors, KiSS-1 levels are lower than ERα-negative tumors (22, 23). In contrast, Jarzabek and colleagues demonstrated that ERα-positive breast tumors express higher levels of both KiSS-1 and KiSS-1R than ERα-negative tumors (24).

Recently, *KiSS-1R* has been documented to induce invasion of triple negative breast cancer (TNBC) cells which lack ERα, progesterone receptor, and human epidermal growth factor receptor 2; also, *KiSS-1* mRNA and *KiSS-1R* mRNA and protein were found to be upregulated in TNBC tissues as compared to normal breast tissue (25).

So far, it is not possible to assign a clear role to KiSS-1/KiSS-1R system in regulating the progression of ERα-positive and ERα-negative breast tumors as the complex cross-talk between KiSS-1 expression and signaling pathways regulated by ERα deserve further investigation.

### Studies on Other Tumors

Since the development of metastases is one of the most dangerous complications of solid tumors, many efforts are conducted to discover and characterize new potential anti-metastatic targets.

Colorectal cancer is identified as one of the most frequent and deadly types of cancer; it represents the second most common tumor among women and the third most common among men (26).

A major complication of CRC is disease progression *via* liver metastases.

Metastases from CRC are strictly associated with matrix metalloproteinase (MMP)-9 expressions (27, 28).

The analysis of KiSS-1 and KiSS-1R expression in colorectal liver metastases showed the existence of a correlation with the patients’ prognosis. CRC tissues with low levels of KiSS-1 express high levels of MMP-9 and metastasize more frequently to distant sites (29).

Accordingly, Chen et al. showed that *KiSS-1* gene represses the metastatic potential of CRC cells by inhibiting the expression of MMP-9. Overexpression of *KiSS-1* suppressed the proliferation and the invasiveness of HCT-119 CRC cells and enhanced their apoptosis by reducing the expression of MMP-9 through blocking PI3K/Akt/NF-κB pathway (30).

The analysis of KiSS-1 and KiSS-1R expression in normal and malignant tissue samples from 111 patients with colorectal adenocarcinoma showed that KiSS-1 expression levels were much higher in the normal than in the malignant colonic mucosa (31).

Regarding malignant tissues, it has been shown that the expression level of KiSS-1 had a negative correlation with Dukes staging, TNM (tumor, lymph node, and metastasis) staging, tumor size, and lymph node involvement. Reduction of KiSS-1R was also linked to poor prognosis of the patients (32).

Studies of comparison of the miRNA expression profiles in CRC tissues and hepatic metastasis revealed that down-regulation of miR-199b associates with distant metastasis in CRC and a longer median survival. Through human tumor metastasis PCR array, Shen et al. identified *KiSS-1* as one of the downstream targets of SIRT1: silencing of SIRT1 upregulates KiSS-1 expression by enhancing the acetylation of the transcription factor CREB which, in turn, may be activated through its binding to the promoter of *KiSS-1*. Thus, miR-199b regulates SIRT1/CREB/KiSS-1 signaling pathway and might serve as a prognosis marker for patients with CRC (33).

So KiSS-1 and MMP-9 could be considered as prognostic markers in patients with CRC.

An inverse correlation between KiSS-1 and MMP-9 has been demonstrated also in non-small cell lung cancer (NSCLC) patients. It has been reported that *KiSS-1* and MMP-9 mRNA and protein correlate with disease stage, metastasis, and survival of the patients. In particular, KiSS-1 expression was found to be lower in the metastatic tissues as compared to the primary tumors, supporting the notion that *KiSS-1* may be considered as a metastasis suppressor in NSCLC (34).

The potential use of KiSS-1 and KiSS-1R as favorable prognostic markers in NSCLC has been confirmed by Sun et al. in 2013 (35). They analyzed 56 NSCLC specimens divided into low stage (locally advanced) and metastatic (advanced) disease and they found an inverse correlation between KiSS-1 and KiSS-1R expression and NSCLC progression. In particular, they assessed that the expression levels of KiSS-1 and KiSS-1R were lower in cancer tissues compared to normal tissues; moreover, KiSS-1 and KiSS-1R expression was lower in patients with advanced stage compared to patients with low stage of NSCLC. Additionally, they found that cells collected from low stage disease showed high apoptotic ratio and arrest in G1 phase, suggesting a role of the KiSS-1/KiSS-1R system not only in invasion and migration, but also in apoptosis and cell cycle processes.

Furthermore, studies on cisplatin-resistant NSCLC cells demonstrated that overexpression of exogenous *KiSS-1* significantly decreases their invasive capability *in vitro* and *in vivo* (36).

*KiSS-1* has been shown to inhibit the proliferation and invasion also of gastric carcinoma cells *in vitro* and *in vivo* through the downregulation of MMP-9 (37).

A study on 40 gastric cancers divided into two groups according to their high or low *KiSS-1* mRNA expression levels and compared with their adjacent normal gastric mucosa, demonstrated that KiSS-1 may represent an independent prognostic factor for gastric cancer patients. Indeed, the low expression of *KiSS-1* in tumor tissues was documented to correlate with the propensity of gastric cancers to invade, metastasize, and relapse as well as to worse overall and disease-free survival (38). Immunohistochemical analysis of tissue microarrays from 71 patients with gastric cancer revealed a statistically significant reduction of KiSS-1 in lymph node and liver metastases compared with primary tumors (39).

A potential role of the *KiSS-1* gene product, metastin, and its receptor in modulating the biological behavior of thyroid carcinomas has been suggested.

Ringel and co-workers demonstrated that metastin is expressed in normal thyroid and in papillary thyroid carcinomas, while KiSS-1R is overexpressed in papillary thyroid cancer but not in normal thyroid and in follicular adenomas. The expression of KP and its receptor are less common in follicular carcinomas. The authors suggest that the high ability of follicular carcinomas to metastasize is due to their low expression of KiSS-1 product and KiSS-1R. They also show that KiSS-1R activates MAPK, but not Akt in thyroid cancer cells (40). Moreover, KiSS-1 expression is significantly higher in advanced tumors with extra-thyroidal invasion compared to thyroid tumors in the early stages. Decreased expression of KiSS-1R seems to attenuate signaling of the KiSS-1/KiSS-1R system, possibly leading to tumor growth (41).

Another type of cancer in which the expression of *KiSS-1* is correlated with stage and tumor grade is bladder cancer. Sanchez-Carbayo and co-workers found lower levels of *KiSS-1* in bladder cancer tissues as compared to normal counterpart; also, they documented that the loss of *KiSS-1* is associated with bladder cancer progression and clinical outcome (42).

Successively, Cebrian and co-workers identified an epigenetic silencing of *KiSS-1* due to a CpG island hypermethylation near to its promoter region, which correlates with lower levels of *KiSS-1* transcripts in invasive bladder cancer as compared to superficial tumors. These findings highlight the value of *KiSS-1* as a prognostic biomarker (43).

The occurrence of an epigenetic regulation of *KiSS-1* expression which favors bladder cancer invasion was confirmed by Zhang and co-workers. They found that ubiquitin-like with PHD and RING finger domains 1 increase the methylation of CpG nucleotides of *KiSS-1*, thus reducing its expression (44).

Finally, *KiSS-1* overexpression has been documented to regulate apoptosis by increasing caspase 3 and Bax and decreasing Bcl-2 and Bax mRNA levels in human osteosarcoma MG-62 and U2OS cells. In these cell lines, *KiSS-1* overexpression reduces the extent of both cell proliferation and invasiveness (45).

It has been shown that KiSS-1 exerts an anti-metastatic role in human hepatocellular and renal carcinoma by inhibiting metalloproteinase MMP-9 and MMP-2 activity (46, 47).

In human head and neck squamous cell carcinoma (HNSCC) tumors, loss of *KiSS-1* expression has been associated with high metastatic potential compared with non-metastatic tumors (48).

## Role of KiSS-1R in the Acquisition of Drug Resistance

Recent papers attribute to KiSS-1/KiSS-1R complex a diverse function from that observed in other tumor types.

Genetic reconstitution of *KiSS-1* in cisplatin-resistant HNSCC cells has been shown to induce alterations in cisplatin metabolism thus restoring platinum sensitivity (48).

In TNBC, Blake and co-workers documented that KiSS-1R signaling promotes drug resistance in ERα-negative breast cell lines and in TNBC cells by increasing the expression of the efflux drug transporter breast cancer-resistance protein and also by promoting tyrosine kinase (AXL) expression and activity. The authors demonstrated that KiSS-1R activity is necessary to promote drug resistance in ERα-negative breast cell lines and in TNBC cells, since KiSS-1R antagonist restored cell sensitivity to doxorubicin. These findings suggest the possibility to consider KiSS-1R as a possible novel therapeutic target to restore drug sensitivity in patients affected by TNBC (25).

## Role of KiSS-1 in Early Stage of Tumor Development

An additional role of KiSS-1/KiSS-1R was demonstrated by Cho et al. in the early steps of breast cancer development. Using transgenic mice expressing the polyoma middle T antigen under the control of MMTV (mouse mammary tumor virus) long terminal repeat promoter (MMTV-PyMT), the authors found that heterozygous mouse for *KiSS-1 or KiSS-1R* showed delayed hyperplasia, resulting in a late breast cancer initiation, progression, and lung metastasis. Also, they showed that *KiSS-1 and KiSS-1R* silencing in pubertal breast epithelium inhibits mammary gland hyperplasia (49). These findings highlight the role of KiSS-1/KiSS-1R complex in the early phase of breast tumor development.

## KiSS-1 Induces a Dormancy State in Disseminated Tumor Cells

Nash et al. hypothesized that secreted KiSS-1 was able to keep the disseminated melanoma cells in a state of dormancy inducing a suppression of metastatic colonization to multiple organs. The authors showed that mice injected intravenously with cells expressing KiSS-1 without the secretion sequence developed lung metastasis. Also, the mouse survival fell quickly after the interruption of the dormancy and the reactivation of proliferation. Thus, the possibility to keep tumor cells in a dormant state and obtain high survival through the use of exogenous KiSS-1 therapy has been suggested to represent an important goal for the treatment of tumor patients. In addition, the capability of KiSS-1 to maintain the cells in a dormancy state was documented to occur in the absence of its cognate receptor, raising the possibility that additional KiSS-1 receptors and/or paracrine signals exist with the potential to be selectively targeted (50). Similar hypothesis was formulated by Beck et al., suggesting that many tumor cells do not express KiSS-1 receptor, so a paracrine control may exist between tumor cells expressing KiSS-1 and stromal cells expressing KiSS-1R. KiSS-1 may activate or induce stromal cells to produce secreted factors in the surrounding extracellular milieu that, directly or indirectly, elicit dormancy in metastatic cells (51).

## Conclusion

Although initially the KiSS-1/KiSS-1R complex was described to be involved in the onset of puberty, sexual maturity, and pregnancy through direct regulation of the hormone releasing the gonadotropin produced by the hypothalamus, other multiple roles have been proposed for this complex in the tumor development process.

Metastasis is a main cause of death in cancer patients and involves a multi-step process encompassing detachment of cancer cells from a primary tumor, invasion of adjacent tissue, transvasation of blood vessels, and spread through circulation, distant organs colonization. *KiSS-1* has been described as a gene suppressor of metastasis in melanoma and more recently in other types of cancer, such as breast cancer, CRC, lung, thyroid, bladder, gastric, and other cancers.

In this mini-review, we highlighted other roles of the KiSS-1/KiSS-1R complex in addition to the role of suppressor gene of metastases. One of the main functions that have been found is the involvement of this complex in drug resistance. The development of drug resistance is still one of the main obstacles in effective cancer treatment. Therefore, there is still an unmet need to identify the molecules that could be targeted in order to overcome resistance in the treatment of tumors and the KiSS-1/KiSS-1R complex may represent a potential target for the treatment of drug-resistant tumors. In fact, it has been described that the KiSS-1/KiSS-1R system is involved in the sensitivity to traditional drugs, since the reconstitution of *KiSS-1* in cisplatin-resistant head and neck cancer cells restores platinum sensitivity; in addition, KiSS-1R has been reported to be involved in the process of drug resistance in TNBC.

Furthermore, a potential role of the KiSS-1/KiSS-1R complex has been proposed in the early stage of breast cancer development so this complex is not only involved in the advanced stages of the tumor, but could also represent a good target to hit tumors at an initial and final stage of development.

Finally, a role played by KiSS-1 in the dormancy of disseminated tumor cells and in the suppression of multiple organ metastases was described, representing the possibility of maintaining the tumor in an asymptomatic state. This could be a valid method to intervene on the block of metastatic development through the treatment with KiSS-1 that inducing tumor cell dormancy could block the metastatic process. The perspective that KiSS-1 can be used in clinical treatment is really favorable because KiSS-1 is a natural product that can be administered at high levels to humans without toxicity.

## Author Contributions

MM: conception of the work. FF and MM: performed extensive literature search. MM and FF: manuscript drafting. MM and MC: critical revision of the work. MM: final version approval.

## Conflict of Interest Statement

The authors declare that the research was conducted in the absence of any commercial or financial relationships that could be construed as a potential conflict of interest. The reviewers FM, AM and handling Editor declared their shared affiliation.
